# Protocol for profiling circulating T cell subsets, their homing and regulatory markers, in pediatric samples using a 4-laser BD LSRII

**DOI:** 10.1016/j.xpro.2026.104701

**Published:** 2026-07-10

**Authors:** Tomas Thon, Jan Svoboda, Zaneta Slavickova, Eliska Kopelentova, Dagmar Srutkova, Stepan Coufal, Zuzana Reiss, Miloslav Kverka, Stepanka Capkova, Jana Cadova, Anna Sediva, Helena Tlaskalova-Hogenova, Andrea Polouckova, Zuzana Jiraskova Zakostelska

**Affiliations:** 1 Laboratory of Cellular and Molecular Immunology, Institute of Microbiology of the Czech Academy of Sciences, Prague, Czech Republic; 2 Cytometry and Microscopy Core Facility, Institute of Microbiology of the Czech Academy of Sciences, Prague, Czech Republic; 3 Department of Immunology, 2nd Faculty of Medicine, Charles University and Motol and Homolka University Hospital, Prague, Czech Republic; 4 Laboratory of Gnotobiology, Institute of Microbiology of the Czech Academy of Sciences, Novy Hradek, Czech Republic; 5 Department of Paediatric Dermatology, Motol and Homolka University Hospital, Prague, Czech Republic; 6 Institute of Clinical Immunology and Allergology, First Faculty of Medicine, Charles University and General University Hospital in Prague, Prague, Czech Republic

**Keywords:** Cell isolation, Flow Cytometry, Immunology

## Abstract

Here, we present a protocol for profiling T cell subsets, regulatory phenotypes, and gut- and skin-homing markers of unstimulated cells or those stimulated with staphylococcal enterotoxin B (SEB), lipopolysaccharide (LPS), or zymosan (ZYM). We describe steps for isolation, cryopreservation, thawing, stimulation, and multiparameter flow cytometric analysis of human peripheral blood mononuclear cells (PBMCs). Use of the multicolored panel detailed in this protocol allows full utilization of the valuable samples with the minimal possible volume of collected pediatric blood.

For complete details on the use and execution of this protocol, please refer to Thon et al.^1^

## Highlights

• Steps for isolation and cryopreservation of PBMCs from pediatric blood
• 4-laser BD LSRII flow cytometry for T cell subsets and gut/skin homing markers
• Identification of regulatory T cells in pediatric PBMCs
• Instructions for PBMC stimulation with SEB, LPS, and ZYM

## Abstract (editor highlights)

Publisher’s note: Undertaking any experimental protocol requires adherence to local institutional guidelines for laboratory safety and ethics.

## Graphical abstract

**Figure:**
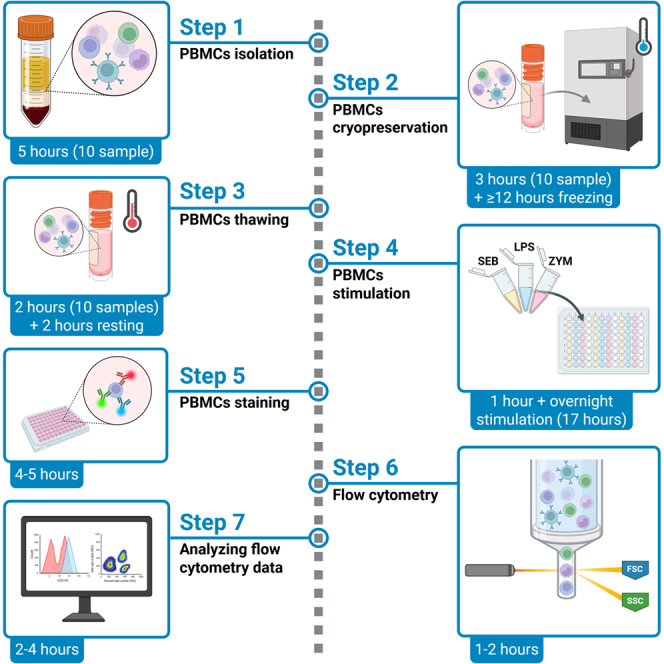

## Before you begin

Peripheral blood mononuclear cells (PBMCs) represent a heterogeneous population of immune cells that can be used to evaluate immune responses based on function and phenotype.^2^ Analyzing T-cell subsets requires careful integration of stimulation conditions with multiparameter flow cytometry to resolve immune activation, homing, and regulatory markers within a single assay.

Here, we describe a workflow combining PBMCs isolation, cryopreservation, thawing, stimulation with superantigenic staphylococcal enterotoxin B (SEB), bacterial endotoxin lipopolysaccharide (LPS), and fungal cell wall component zymosan (ZYM) stimuli, and multicolor flow cytometric analysis on a device with a limited number of lasers. These stimuli were selected to model distinct modes of immune activation. The protocol is optimized for use with cryopreserved PBMCs from pediatric patients with atopic dermatitis and food allergy but can be applied to PBMCs from other human donors with minor adjustments.

The staining strategy integrates extracellular and intracellular marker detection using a conventional flow cytometer with only a 4-laser configuration and therefore requires careful optimization of antibody panels, controls, and compensation. Appropriate use of fluorescence-minus-one (FMO) and single-stain (SS) controls is essential for accurate gating and data interpretation.^3^ Although this protocol was developed using a BD LSRII flow cytometer, it may be adapted to other instruments with comparable laser and detector configurations by adjusting fluorochrome selection and panel design according to instrument capabilities.

### Innovation

This protocol integrates PBMCs isolation, long-term cryopreservation, and multiparameter flow cytometric analysis into a single, standardized workflow optimized for characterizing human T-cell subsets. The panel combines markers for T-cell lineage, differentiation, regulatory phenotype, and homing status enabling detailed analysis of multiple T-cell subpopulations within a single run on a conventional 4-laser flow cytometer. By including unstimulated PBMCs together with SEB, LPS, and ZYM stimulated PBMCs within the same experimental design, the protocol enables comparative assessment of T-cell responses to distinct immune stimuli using cryopreserved clinical samples.

### Institutional permissions

This study was approved by the Ethics Committee of Motol University Hospital (approval date 22 May 2019). The children’s legal representatives gave their written consent for their children to take part in this study. Researchers who apply this protocol should obtain approval from the relevant institutional ethics committee and secure all required permissions before collecting and processing human samples.

## Key resources table

**Table:** 

REAGENT or RESOURCE	SOURCE	IDENTIFIER
**Antibodies**		

Mouse monoclonal anti-human CD3, Alexa Fluor™ 488 conjugated (diluted 1:50)	Thermo Fisher Scientific	Cat#53-0037-42; RRID:AB_1907370; clone OKT3
Mouse monoclonal anti-human CD4, violetFluor™ 450 conjugated (diluted 1:20)	Cell Signaling Technology	Cat#26755S; RRID:AB_2798930; clone RPA-T4
Mouse monoclonal anti-human CD8, redFluor™ 710 conjugated (diluted 1:40)	Abcam	Cat#ab241937; clone RPA-T8
Mouse monoclonal anti-human CD25, PE-eFluor™ 610 conjugated (diluted 1:10)	Thermo Fisher Scientific	Cat#61-0257-42; RRID:AB_2574544; clone CD25-4E3
Mouse monoclonal anti-human FoxP3, APC conjugated (diluted 1:10)	Thermo Fisher Scientific	Cat#17-4777-42; RRID:AB_10804651; clone 236A/E7
Rat monoclonal anti-human CCR7, PerCP-eFluor™ 710 conjugated (diluted 1:10)	Thermo Fisher Scientific	Cat#46-1979-42; RRID:AB_10853814; clone 3D12
Mouse monoclonal anti-human CD45RA, StarBright Violet 710 conjugated (diluted 1:20)	Bio-Rad	Cat#MCA88SBV710; RRID:AB_3101672; clone F8-11-13
Mouse monoclonal anti-human CD45RO, Super Bright™ 600 conjugated (diluted 1:20)	Thermo Fisher Scientific	Cat#63-0457-42; RRID:AB_2662466; clone UCHL1
Mouse monoclonal anti-human CCR9, Brilliant Violet 510 conjugated (diluted 1:10)	BD Biosciences	Cat#752588; RRID:AB_2917575; clone C9Mab-1
Rat monoclonal anti-human CLA, PE/Cyanine7 conjugated (diluted 1:20)	BioLegend	Cat#321316; RRID:AB_2565768; clone HECA-452
Mouse monoclonal anti-human CD14, APC-eFluor™ 780 conjugated (diluted 1:20)	Thermo Fisher Scientific	Cat#47-0149-41; RRID:AB_1834359; clone 61D3
Mouse monoclonal anti-human CD16, APC-eFluor™ 780 conjugated (diluted 1:20)	Thermo Fisher Scientific	Cat#47-0166-42; RRID:AB_2848353; clone 3G8
Mouse monoclonal anti-human CD19, APC-eFluor™ 780 conjugated (diluted 1:20)	Thermo Fisher Scientific	Cat#47-0199-42; RRID:AB_1582230; clone HIB19
Mouse monoclonal anti-human CD56, APC-eFluor™ 780 conjugated (diluted 1:20)	Thermo Fisher Scientific	Cat#47-0567-42; RRID:AB_10854573; clone CMSSB
Fc Receptor Binding Inhibitor Polyclonal Antibody	Thermo Fisher Scientific	Cat#14-9161-73; RRID:AB_468582

**Biological samples**		

Blood samples	this study	N/A

**Chemicals, peptides, and recombinant proteins**		

Phosphate Buffered Saline (PBS), tablets	MP Biomedicals	Cat#0928103-CF
Ficoll-Paque™ PLUS density gradient media	Cytiva	Cat#17144002
RPMI-1640 Medium (Sigma-Aldrich)	Merck KGaA	Cat#R0883
HyClone Research Grade Fetal Bovine Serum, South American Origin (FBS)	Cytiva	Cat#SV30160.03
Dimethyl sulfoxide Hybri-Max™ (DMSO)	Sigma Aldrich, Merck	Cat#D2650
Antibiotic Antimycotic Solution (100×), Stabilized (ATB)	Merck KGaA	Cat#A5955
L-Glutamine	Merck KGaA	Cat#G6392
Staphylococcal enterotoxin B from Staphylococcus aureus (SEB)	Sigma Aldrich, Merck	Cat#S4881
Zymosan (ZYM)	InvivoGen	Cat#tlrl-zyn
LPS from Salmonella abortus equi S-form (TLRGRADE®) (Ready-to-Use)	Enzo Life Sciences	Cat#ALX-581-009
Fixable Viability Dye eFluor™ 780 (diluted 1:200)	Thermo Fisher Scientific, Invitrogen™, eBioscience™	Cat#65-0865-18
Foxp3/Transcription Factor Staining Buffer Set	Thermo Fisher Scientific, Invitrogen™, eBioscience™	Cat#00-5523-00

**Software and algorithms**		

FlowJo, version 10.1.0.0	BD Biosciences	https://www.flowjo.com
BD FACSDiva™ software, version 8.0.1	BD Biosciences	
GraphPad Prism, version 8.4.3	GraphPad Software	https://www.graphpad.com

**Other**		

VACUETTE® lithium heparin blood collection tubes without separation gel 9 mL	Dialab	Cat#455084
EVE™ Automated Cell Counter	NanoEnTek	N/A
Corning® CoolCell™ Freezer Container	Merck KGaA	Cat#CLS432000
Sterile 96-well U-well tissue culture plates	TPP	Cat#TP92097
UltraComp eBeads™ Plus Compensation Beads	Thermo Fisher Scientific, Invitrogen™	Cat#01-3333-42
BD LSRII Flow cytometer with 4 laser excitation lines	BD Biosciences	N/A
BD FACSDiva™ CS&T Research Beads	BD Biosciences	Cat#655051

## Materials and equipment

**Table:** Complete RPMI medium

Reagent	Final concentration	Amount
RPMI	–	44 mL
ATB	1%	0.5 mL
FBS	10%	5 mL
glutamine	1%	0.5 mL

Store at 4 °C no more than 1 week. Warm to 37 °C before use. Ideally, use the same LOT of FBS with low LPS levels for all experiments to minimize variability in immune activation.

### Flow cytometer

The panels used in this protocol were designed for a BD LSR II flow cytometer equipped with the following four lasers in 6-4-5-3 channel configuration: violet 405 nm, blue 488 nm, yellow 561 nm, and red 633 nm lasers.

## Step-by-step method details

### PBMC isolation from whole blood

**Timing: 5 h (10 samples)**

This step describes the isolation of peripheral blood mononuclear cells (PBMCs) from heparinized human whole blood using Ficoll density gradient centrifugation.

***Note:*** PBS used in this protocol does not contain Ca^2+^ or Mg^2+^ ions, as these divalent cations promote cell aggregation, disrupt the density gradient, and induce clumping during staining and flow cytometry acquisition.^4^ Equivalent laboratory-prepared PBS solutions without Ca^2+^ or Mg^2+^ ions may also be used.

***Note:*** Allow heparinized blood to rest at room temperature (20–25 °C) for 4 h before PBMCs isolation. Do not store blood samples at 4 °C prior to PBMC isolation. Heparin is well suited for this protocol because it preserves PBMCs function for the downstream stimulation, whereas other anticoagulants, such as EDTA, may alter cellular responses.^5^ Maintain consistent handling time across samples to minimize variability in activation status and marker expression.

***Note:*** Prepare sterile PBS from commercial tablets in advance. Dissolve 1 PBS tablet per 100 mL of distilled water. Prewarm PBS and RPMI medium to 37 °C before use. Perform all steps under sterile conditions in a biosafety cabinet.

***Note:*** Keep the cells on benchtop for as little time as possible. Never store PBMCs on ice as the T-cells are susceptible to cold. Exposure to ice affects viability as well as the activation status therefore disturbing the analysis.^6^

1. Transfer the heparinized blood (approximately 5 ml) from an individual donor to a new 50 mL tube.
  a. Dilute heparinized blood 1:1 with sterile prewarmed PBS (without Ca^2+^ or Mg^2+^).

***Note:*** Total volume should be approximately 10 mL.

2. Mix by gentle pipetting three times.
3. Add 15 mL of Ficoll-Paque to new 50 mL tube.
4. Gently overlay the diluted blood over Ficoll.
  a. Avoid mixing at the interface.

**CRITICAL:** Add the diluted blood slowly along the inner wall of the tube so that it gently flows over the Ficoll layer. The blood should initially be added dropwise to establish a stable interface. Mixing the two layers will disrupt the density gradient and may result in poor separation of PBMCs during centrifugation.

5. Centrifuge at 700 × g for 30 min at 23 °C with the brake turned off.

**CRITICAL:** Do not use the centrifuge brake during deceleration. Sudden stopping can disrupt the density gradient and mix the phases, leading to poor separation and reduced recovery of mononuclear cells.

**Figure 1:**
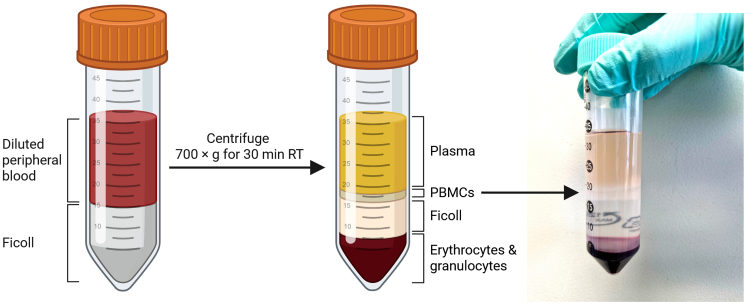
Ficoll density gradient separation of PBMCs Diluted peripheral blood is layered over Ficoll and centrifuged at 700 × g for 30 min at 23 °C. Density-based separation results in plasma (top), PBMCs at the plasma–Ficoll interface, Ficoll, and erythrocytes/granulocytes (bottom). The PBMCs fraction is collected for further analysis.

***Note:*** After centrifugation, several distinct layers should be visible. From top to bottom these are: plasma, a thin opaque whitish ring containing PBMCs at the plasma/Ficoll interface, the transparent Ficoll layer, and the pellet of erythrocytes and granulocytes at the bottom of the tube. Carefully aspirate the PBMCs layer located at the top of the interface between the plasma and Ficoll using a pipette.

6. After the centrifuge stops, immediately collect the mononuclear cells from the plasma/Ficoll interphase and transfer into a new sterile 50 mL tube (Figure 1).
  a. Be sure to aspirate as little Ficoll as possible while collecting the cells.

7. Add approximately 30 mL of prewarmed PBS.
8. Centrifuge at 330 × g for 10 min at 23 °C with the brake turned on, decant the supernatant.
9. Gently resuspend the pellet and adjust the final volume to 1 mL with prewarmed RPMI.
10. Count the cells using Trypan blue exclusion using cell counter.
  a. Proceed with the cryopreservation.

***Note:*** Cell counting and viability assessment may be performed manually using a Bürker chamber or with an equivalent automated cell counter.

### Cryopreservation of freshly isolated PBMCs

**Timing: 3 h (10 samples) plus ≥12 h freezing**

This step describes the cryopreservation of freshly isolated PBMCs for long-term storage.

11. Centrifuge the PBMCs suspension at 330 × g for 10 min at 23 °C with the brake turned on. Decant the supernatant.
12. Meanwhile prepare freezing medium no.1 (40% RPMI, 60% FBS) and freezing medium no.2 (80% FBS, 20% Dimethyl sulfoxide (DMSO)).
  a. Prepare the volume of freezing medium no.1 necessary to adjust the cell concentration to 20x10^6^ cells/mL.
  b. Prepare identical volume of freezing medium no.2. Addition of this solution will adjust the concentration to 10×10^6^ cells/mL.
13. Dilute the cells with freezing medium no.1 to adjust the cell concentration to 20x10^6^ cells/mL.
  a. Mix cells gently by tapping the tube without using a pipette.
14. Slowly drop by drop add an equal volume of freezing medium no.2 while gently swirling the tube to adjust the cell concentration to 10x10^6^ cells/mL.

**CRITICAL:** Add DMSO-containing freezing medium gradually along the wall of the tube while continuously and gently swirling the suspension to ensure even mixing and to minimize osmotic shock, thereby preserving cell viability. Rapid addition of the freezing medium or insufficient mixing may result in reduced post-thaw viability.^7^

**CRITICAL:** Minimize the time cells remain in freezing medium at room temperature (20–25 °C), as prolonged exposure to DMSO reduces cell viability.

15. Incubate for 5 min at room temperature (20–25 °C) to allow equilibration of the freezing medium and reduce osmotic stress prior to freezing.
16. Aliquot the resulting suspension containing the PBMCs into the pre-labeled cryovials (approximately 600-1000 μL per cryotube).

***Note:*** Adjust this volume according to the total number of cells required per sample for all planned experimental conditions, including stimulation conditions, controls, or replicates.

17. Place cryovials into controlled-rate freezing container that has been equilibrated to room temperature (20–25 °C) and transfer the controlled-rate freezing container to a −80 °C freezer for a minimum of 12 h and no more than 48 h.
  a. After that transfer the cryovials to the −150 °C freezer or in liquid nitrogen for long-term storage.

**Pause point:** Cryopreserved PBMCs can be stored long-term at −150 °C freezer or/liquid nitrogen until thawing.

### Thawing and recovery of cryopreserved PBMCs

**Timing: 2 h (for 10 samples) + 2 h resting**

This step describes the thawing of cryopreserved PBMCs, removal of DMSO and cell counting.

***Note:*** Warm up water bath to 37 °C. Warm up RPMI medium as well as complete RPMI medium to 37 °C before use.

18. Remove cryovials containing frozen PBMCs from −150 °C/liquid nitrogen and immediately place them into the 37 °C water bath and thaw the cells for approximately 1-2 min. Remove the cryovials from the water bath when only a small amount of ice remains.
  a. Transfer cryovials from storage to the water bath on dry ice to minimize temperature fluctuations prior to thawing.
  b. Do not allow the cells to thaw completely in the water bath.
19. Immediately transfer the partially thawed cell suspension into a sterile 15 mL tube.
  a. Pipette slowly using a 1 mL tip to minimize shear stress and preserve cell viability.
20. Slowly drop by drop add 8 mL of pre-warmed RPMI medium into the tube to wash out the DMSO from the cells.
  a. Use a portion of the medium to wash out the cryovial and transfer this volume into the same 15 mL tube.
  b. Centrifuge at 300 × g for 5 min at 23 °C with the brake turned on.
  c. Carefully decant the supernatant.
  d. Repeat the washing step once more for a total of two washes.
21. Resuspend the PBMCs pellet in 1 mL of pre-warmed complete RPMI medium.
22. Determine cell concentration and viability using Trypan blue exclusion.

***Note:*** Our typical viability of thawed PBMCs ranges from 70% to 90%.

23. Adjust the concentration to 5 × 10^6^ viable cells/mL using complete RPMI medium [troubleshooting 1].
24. Plate 100 μL per well (5 × 10^5^ cells per well) into a sterile 96-well U-bottom tissue culture plate according to your layout (see Table 1).
  a. Prepare a pooled control cell suspension from the remaining cells and plate 100 μL per well into the same 96-well U-bottom tissue culture plate according to the experimental layout (see Table 1).

**Table 1:** Example 96-well plate layout

	1	2	3	4	5	6	7	8	9	10	11	12
A	Sample A	Sample A	Sample A	–	Sample A	–	Sample F	Sample F	Sample F	–	Sample F	–
	SEB	ZYM	LPS	–	UNSTIM	–	SEB	ZYM	LPS	–	UNSTIM	–
B	Sample B	Sample B	Sample B	–	Sample B	–	Sample G	Sample G	Sample G	–	Sample G	–
	SEB	ZYM	LPS	–	UNSTIM	–	SEB	ZYM	LPS	–	UNSTIM	–
C	Sample C	Sample C	Sample C	–	Sample C	–	Sample H	Sample H	Sample H	–	Sample H	–
	SEB	ZYM	LPS	–	UNSTIM	–	SEB	ZYM	LPS	–	UNSTIM	–
D	Sample D	Sample D	Sample D	–	Sample D	–	Sample I	Sample I	Sample I	–	Sample I	–
	SEB	ZYM	LPS	–	UNSTIM	–	SEB	ZYM	LPS	–	UNSTIM	–
E	Sample E	Sample E	Sample E	–	Sample E	–	Sample J	Sample J	Sample J	–	Sample J	–
	SEB	ZYM	LPS	–	UNSTIM	–	SEB	ZYM	LPS	–	UNSTIM	–
F	POOL	POOL	POOL	POOL	POOL	POOL	POOL	POOL	POOL	POOL	POOL	POOL
	UNSTIM	UNSTIM	UNSTIM	UNSTIM	UNSTIM	UNSTIM	UNSTIM	UNSTIM	UNSTIM	UNSTIM	UNSTIM	UNSTIM
G	POOL	POOL	POOL	POOL	POOL	POOL	POOL	POOL	POOL	POOL	POOL	POOL
	UNSTIM	UNSTIM	UNSTIM	UNSTIM	UNSTIM	UNSTIM	UNSTIM	UNSTIM	UNSTIM	UNSTIM	UNSTIM	UNSTIM
H	–	–	–	–	–	–	–	–	–	–	–	POOL
	–	–	–	–	–	–	–	–	–	–	–	UNSTIM

Individual PBMCs samples (A–J) are plated under four conditions: stimulation with Staphylococcal enterotoxin B (SEB), zymosan (ZYM), lipopolysaccharide (LPS), or unstimulated (UNSTIM). Pooled control cell suspensions (POOL) were created by combining remaining PBMCs from individual samples and were plated as unstimulated controls.
25. Let the cells rest for 2 h in a humidified incubator at 37 °C and 5% CO_2_ prior to stimulation.

### PBMC stimulation with SEB, LPS, and ZYM

**Timing: 30 min + overnight stimulation (17 h)**

This step describes the in vitro stimulation of thawed PBMCs using SEB, LPS, and ZYM.

***Note:*** When using different stimuli, test the optimal concentration, as excessive stimulation may induce cell death, while insufficient stimulation may result in no detectable response.

26. Prepare stimulation reagents in complete RPMI medium according to the desired final concentrations:
  a. SEB: prepare at 2 μg/mL to achieve a final concentration of 1 μg/mL after addition to cells [troubleshooting 2].
    **CRITICAL:** Staphylococcal enterotoxin B (SEB) is a thermostable toxin.^8^ Handling of SEB may require specific institutional or national approval and biosafety authorization. Prepare SEB solutions carefully. Dispose of unused SEB solutions and all materials that have come into contact with SEB in accordance with institutional and national biosafety guidelines.
  b. LPS: prepare at 0.2 μg/mL to achieve a final concentration of 0.1 μg/mL after addition to cells [troubleshooting 2].
  c. ZYM: prepare at 100 μg/mL to achieve a final concentration of 50 μg/mL after addition to cells [troubleshooting 2].

27. Add 100 μL of stimulation medium containing SEB, LPS, or ZYM to the appropriate wells according to your layout (see Table 1).
28. For unstimulated controls (UNSTIM), add 100 μL of complete RPMI medium without stimulus (see Table 1).
29. Fill empty wells on the plate with sterile PBS or sterile water to minimize evaporation.
30. Incubate cells for 17 h in a humidified incubator at 37 °C and 5% CO_2_.

### Flow cytometric staining of unstimulated and stimulated PBMCs

**Timing: 3–4 h**

This step describes viability staining, fixation/permeabilization, intracellular staining, and flow cytometric acquisition of unstimulated and stimulated PBMCs for the analysis of T-cell subsets, regulatory markers, activation markers and gut and skin homing phenotypes.

***Note:*** Prepare the FoxP3/Transcription Factor Fixation/Permeabilization working solution (Step 40) and 1× Permeabilization/Wash buffer (PERM) (Steps 41 to 46) according to the manufacturer’s instructions using the Foxp3/Transcription Factor Staining Buffer Set by diluting Permeabilization Buffer (10X) with distilled water.

31. Centrifuge the 96-well plate at 300 × g for 5 min at 4 °C. Confirm the presence of a visible cell pellet.

***Note:*** If no visible pellet is present, centrifuge the plate again under the same conditions.

32. To remove the supernatant, flick the plate by quickly and firmly inverting it over a waste container using a swift, single-motion wrist snap. Ensure the motion is strictly vertical to prevent cross-contamination. Follow this by blotting the inverted plate on paper towels to remove any residual liquid.
  a. Repeat this process whenever decanting the supernatant.
33. Wash wells containing the cells with 170 μL of PBS and centrifuge at 300 × *g* for 5 min at 4 °C. Decant the supernatant.
34. Gently resuspend the pellet by brief low-speed vortexing and add 20 μL of diluted human Fc receptor blocking reagent (1:200 in PBS).
  a. Incubate for 20 min on ice.
35. During the incubation, prepare fluorochrome-conjugated antibodies for extracellular staining in PBS according to the experimental design (see Table 2).
  a. Prepare a complete antibody master mix (MIX I) for extracellular multicolor staining of PBMCs samples.
  b. Prepare fluorescence-minus-one (FMO) controls for extracellular staining by omitting one antibody at a time.
  c. Prepare individual single-stain (SS) antibody solutions for extracellular staining of pooled control cells and compensation beads.
    i. For the dump channel SS control, use only one antibody with the appropriate fluorochrome.

**Table 2:** Antibodies, dilution ratios, staining type, lasers, and filters: LP-Long Pass, BP-Band pass

Name	Dilution	Staining	Lasers nm	Filters nm
CD3, Alexa Fluor 488	1:50	Extracellular	488	505 LP, 525/50 BP
CD4, violetFluor 450	1:20	Extracellular	405	450/50 BP
CD8, redFluor 710	1:40	Extracellular	633	710 LP, 730/45 BP
CD25, PE-eFluor 610	1:10	Extracellular	561	600 LP, 610/20 BP
CCR7, PerCP-eFluor 710	1:10	Extracellular	488	650 LP, 712/21 BP
CCR9, Brilliant Violet 510	1:10	Extracellular	405	505 LP, 530/30 BP
CLA, PE/Cyanine7	1:20	Extracellular	561	735 LP, 780/60 BP
CD45RA, StarBright Violet 710	1:20	Extracellular	405	690 LP, 705/20 BP
CD45RO, Super Bright 600	1:20	Extracellular	405	595 LP, 605/12 BP
CD14, APC-eFluor™ 780 (dump channel)	1:20	Extracellular	633	755 LP, 780/60 BP
CD16, APC-eFluor™ 780 (dump channel)	1:20	Extracellular	633	755 LP, 780/60 BP
CD19, APC-eFluor™ 780 (dump channel)	1:20	Extracellular	633	755 LP, 780/60 BP
CD56, APC-eFluor™ 780 (dump channel)	1:20	Extracellular	633	755 LP, 780/60 BP
Fixable Viability Dye, eFluor 780	1:200	Extracellular	633	755 LP, 780/60 BP
FoxP3, APC	1:10	Intracellular	633	675/40 BP

Antibody dilution ratios were optimized by titration experiments.

***Note:*** Handle fluorochrome-conjugated antibodies under dim light conditions. Fluorochromes are light-sensitive and may undergo photobleaching upon exposure to ambient light leading to reduced fluorescence intensity and decreased signal stability.

**CRITICAL:** At this stage, use only antibodies for **EXTRACELLULAR** (surface) staining (see Table 2). Do not include intracellular antibodies, which must be applied after fixation/permeabilization (Step 40). This also applies to the preparation of FMO and SS controls.

***Note:*** Fluorescence-minus-one (FMO) controls contain all antibodies in the panel except the one of interest and are used to define gating boundaries for dim or overlapping populations. FMO controls account for fluorescence spillover and background signal in multicolor panels and are essential for accurate identification of positive and negative cell populations. FMO controls should be prepared using cells.

***Note:*** Single-stain (SS) controls contain only one fluorochrome-conjugated antibody and are used to calculate compensation between fluorescence channels. SS controls are used to correct for spectral overlap between fluorochromes during data acquisition. SS controls may be prepared using either cells or compensation beads.

***Note:*** A dump channel refers to the use of multiple antibodies conjugated to the same fluorochrome to collectively exclude unwanted cell populations during analysis. In this protocol, CD14, CD16, CD19, and CD56 are combined in a single channel to identify and remove monocytes, NK cells, and B cells from the analysis, thereby enriching for T-cell populations and improving gating specificity (see Table 2).

36. Wash cells with 170 μL of PBS and centrifuge at 300 × *g* for 5 min at 4 °C. Decant the supernatant.
37. Add 20 μL of MIX I for extracellular staining to the wells containing PBMCs samples (rows A to E) [troubleshooting 3].
  a. Reference control: Add 20 μL of MIX I for extracellular staining to the well F12 containing pooled cells (see Table 3; Table 4).

**Table 3:** Overview of flow cytometry controls used in the protocol

Control type	Material	Purpose	Plate location	Staining
Reference control	Pooled cells	Reference multicolor staining	F12	MIX I + MIX II
FMO controls	Pooled cells	Gate definition for dim/overlapping populations	F1–F11	All antibodies except one
SS controls (cells)	Pooled cells	Visualization of staining patterns	G1–G11	Single antibody
SS controls (beads)	Compensation beads	Compensation calculation	H1–H11	Single antibody
Native/FVD control	Pooled cells	Viability dye control	G12	FVD only

**Table 4:** Example 96-well plate layout for multicolor staining and single-stain controls

	1	2	3	4	5	6	7	8	9	10	11	12
A	Sample A	Sample A	Sample A	–	Sample A	–	Sample F	Sample F	Sample F	–	Sample F	–
	MIX	MIX	MIX	–	MIX	–	MIX	MIX	MIX	–	MIX	–
B	Sample B	Sample B	Sample B	–	Sample B	–	Sample G	Sample G	Sample G	–	Sample G	–
	MIX	MIX	MIX	–	MIX	–	MIX	MIX	MIX	–	MIX	–
C	Sample C	Sample C	Sample C	–	Sample C	–	Sample H	Sample H	Sample H	–	Sample H	–
	MIX	MIX	MIX	–	MIX	–	MIX	MIX	MIX	–	MIX	–
D	Sample D	Sample D	Sample D	–	Sample D	–	Sample I	Sample I	Sample I	–	Sample I	–
	MIX	MIX	MIX	–	MIX	–	MIX	MIX	MIX	–	MIX	–
E	Sample E	Sample E	Sample E	–	Sample E	–	Sample J	Sample J	Sample J	–	Sample J	–
	MIX	MIX	MIX	–	MIX	–	MIX	MIX	MIX	–	MIX	–
F	POOL	POOL	POOL	POOL	POOL	POOL	POOL	POOL	POOL	POOL	POOL	POOL
	FMO: CD3 Alexa Fluor 488	FMO: CD4 violetFluor 450	FMO: CD8 redFluor 710	FMO: CD25 PE-eFluor 610	FMO: CCR7 PerCP-eFluor	FMO: CCR9 Brilliant Violet 510	FMO: CLA PE/Cyanine7	FMO: CD45RA StarBright Violet 710	FMO: CD45RO Super Bright 600	FMO: FoxP3 APC	FMO: DUMP APC-eFluor™ 780	MIX (Reference control)
G	POOL	POOL	POOL	POOL	POOL	POOL	POOL	POOL	POOL	POOL	POOL	POOL
	SS: CD3 Alexa Fluor 488	SS: CD4 violetFluor 450	SS: CD8 redFluor 710	SS: CD25 PE-eFluor 610	SS: CCR7 PerCP-eFluor	SS: CCR9 Brilliant Violet 510	SS: CLA PE/Cyanine7	SS: CD45RA StarBright Violet 710	SS: CD45RO Super Bright 600	SS: FoxP3 APC	SS: DUMP APC-eFluor™ 780	Native control (SS FVD)
H	BEADS	BEADS	BEADS	BEADS	BEADS	BEADS	BEADS	BEADS	BEADS	BEADS	BEADS	–
	SS: CD3 Alexa Fluor 488	SS: CD4 violetFluor 450	SS: CD8 redFluor 710	SS: CD25 PE-eFluor 610	SS: CCR7 PerCP-eFluor	SS: CCR9 Brilliant Violet 510	SS: CLA PE/Cyanine7	SS: CD45RA StarBright Violet 710	SS: CD45RO Super Bright 600	SS: FoxP3 APC	SS: DUMP APC-eFluor™ 780	–

Individual PBMCs samples (A–J) are stained with a complete antibody master mixes (MIX I and MIX II). Pooled control cells (POOL) are used to generate fluorescence-minus-one (FMO) controls (F1–F11) and single-stain (SS) controls (G1–G11) using the appropriate antibody mixtures. Well F12 contains pooled cells stained with the complete antibody master mix (MIX I and II) and serves as a staining reference control. Well G12 contains pooled cells stained only with the fixable viability dye (FVD) and functions as a single-stain control for FVD. Compensation beads (BEADS) were stained with individual single-stain (SS) fluorochrome-conjugated antibodies for compensation (H1–H11). The dump channel consists of CD14, CD16, CD19, and CD56 conjugated to APC-eFluor™ 780.
  b. FMO controls: Add 20 μL of the appropriate FMO antibody mixture (all antibodies except the one of interest) for extracellular staining to the designated pooled control cell wells according to the layout (wells F1 to F11) (see Table 3; Table 4).
  c. SS controls: Add 20 μL of the appropriate single-stain antibody solution for extracellular staining to the designated pooled control cell wells (for visualization of staining patterns) (wells G1 to G11 except G10, which is the SS control for intracellular staining) (see Table 3; Table 4).
  d. Native control: Add 20 μL of diluted Fixable Viability Dye (FVD) (1:200) in PBS to the well G12 (see Table 3; Table 4). The well G12 serves as a functional single-stain control for FVD.
38. Incubate for 20–30 min on ice in the dark.
39. Wash cells with 170 μL of PBS and centrifuge at 300 × *g* for 5 min at 4 °C. Decant the supernatant.
40. Resuspend cells in 140 μL of Foxp3/Transcription Factor Fixation/Permeabilization working solution.
  a. Incubate for 45 min on ice in the dark.
41. During the incubation, prepare fluorochrome-conjugated antibodies for intracellular (nuclear) staining (MIX II) in 1× Permeabilization/Wash buffer (PERM).

***Note:*** As FoxP3 is the only intracellular marker in MIX II, the same mix can be used for all relevant wells: wells with samples, the reference control, the appropriate FMO control, and FoxP3 SS control.

42. Wash cells with 170 μL of PERM and centrifuge at 350 × *g* for 5 min at 4 °C. Decant the supernatant.
43. Add 25 μL of compensation beads to designated empty wells according to the plate layout (wells H1 to H11) (see Table 3; Table 4).

***Note:*** Vortex compensation beads thoroughly before use to ensure uniform suspension. Use compensation beads compatible with the antibody species and isotype, as binding efficiency may vary depending on antibody characteristics and fluorochrome conjugates.

44. Add 20 μL of MIX II for the intracellular staining to the wells containing PBMCs samples (rows A to E) [troubleshooting 3].
  a. Reference control: Add 20 μL of MIX II to the well F12 containing pooled cells (see Table 4).
  b. FMO controls: Add 20 μL of MIX II to the wells with the appropriate FMO controls (wells F1 to F11 except F10, which is the FMO for FoxP3) (see Table 4).
  c. SS control: Add 20 μL of MIX II to the appropriate SS control (G10) (see Table 4).
45. Add 20 μL of the appropriate extracellular SS solution prepared at Step 35 (and already used for SS controls with pooled cells) to wells containing compensation beads according to the layout (wells H1 to H11 except H10, which is the SS control for FoxP3).
  a. For the SS control of FoxP3 (well H10), use 20 μL of the MIX II solution prepared at Step 41 (see Table 4).
46. Incubate for 20–30 min on ice in the dark.
47. Wash twice with 170 μL of PERM and centrifuge at 350 × *g* for 5 min at 4 °C. Discard the supernatant.
48. Resuspend stained cells and beads in 100 μL of PBS and measure by flow cytometry.

### Sample acquisition on a 4-laser BD LSRII flow cytometer

**Timing: 2–4 h**

The next step describes acquisition of flow cytometry data using a 4-laser BD LSRII flow cytometer equipped with a high-throughput sampler and fluorescent channels capable of acquiring listed fluorochromes. Data acquisition is performed using BD FACSDiva software (version 8.0.1).

***Alternatives:*** Depending on availability, data may be acquired on other flow cytometers with comparable laser and channel configurations. Recommended excitation lines for this antibody panel are 405 nm, 488 nm, 561 nm, and 633 nm.

**CRITICAL:** Ensure that selected fluorochromes are compatible with the specific laser and detector configuration of the flow cytometer. Antibody panels may require adjustment depending on instrument capabilities.

49. Before acquiring data, use the BD CS&T daily QC beads (BD FACSDiva™ CS&T Research Beads or equivalent) to perform instrument quality control and standardization. Verify that detector voltages are appropriately set for each fluorescence channel to avoid signal saturation or insufficient resolution^9^ [troubleshooting 4 and 5].
50. Use a full antibody mix for each panel (well F12; Table 4) and check that all fluorescence channels are within the detection range (e.g., none of the fluorescent populations are off the scale, excessively bright) [troubleshooting 4 and 5].

***Note:*** In case of too-bright populations and cytometers with photomultiplier tube detectors (PMTs), it is recommended to decrease the antibody concentration rather than substantially reducing detector voltage, as excessive voltage reduction may compromise signal resolution.

51. Use SS controls to set up an appropriate level of compensation and unstained samples to define the baseline level of autofluorescence for all fluorochromes on the flow cytometry machine used [troubleshooting 4 and 5].

***Note:*** All cross-talk/fluorescence compensation steps were performed off-line, after the data acquisition-step during data-evaluation and analysis.

***Note:*** In this protocol, compensation is calculated using bead-based SS controls. Cell-based SS controls are included to verify that each antibody stains the expected cell population and to assess staining patterns on cells. FMO controls are analyzed on cells only.

**CRITICAL:** When using compensation controls, ensure that the negative population matches the corresponding positive population within each control sample. Bead-based and cell-based SS controls may both be used; however, compensation must be calculated using the appropriate local negative population for each control. Use either cell-based or bead-based controls. Note that FVD must be compensated using cell-based SS controls.

***Note:*** Always use the same conjugated antibody for samples and SS controls. Fluorescence properties of tandem dyes and other fluorophore-antibody conjugates may vary depending on fluorochrome stability, degree of labeling, storage conditions, and antibody lot, which can affect spectral spillover and compensation accuracy – e.g. PE-Cy7 fluorochrome on a fresh anti-CD3 MAb may exert different spectral properties from PE-Cy7 on an older anti-CD4 MAb.^10^

52. Acquire a sufficient number of live cells per sample at a flow rate that maintains stable acquisition (typically ≤8,000 events/s, depending on instrument configuration).

***Note:*** For optimal analysis of rare cell subsets (typically representing <1% of live cells), we recommend acquisition of 5–10 × 10^5^ live cells to ensure sufficient target population counts for reliable analysis. For more abundant subsets, 2–5 × 10^5^ live cells are sufficient depending on the frequency of the population of interest. As a general guideline, the total number of acquired events should be adjusted to obtain an adequate number of events within the target population.

***Note:*** The recommended flow rate depends on the sample purity and instrument configuration - where available observe the ratio of flow rate to electronic abort rate and maintain electronic abort rate below 10% of total events.^11^^,^^12^

### Gating strategy and data analysis

**Timing: 1–2 h**

**Figure 2:**
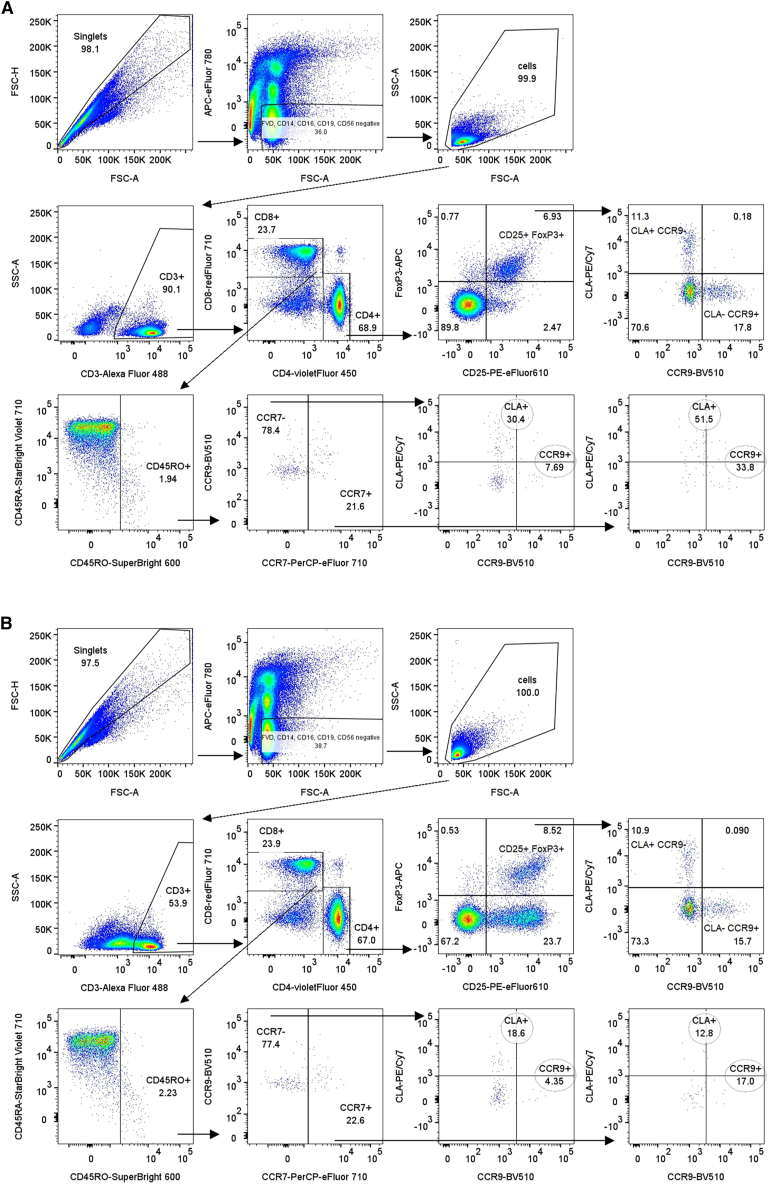
Sequential gating strategy for identification of T-cell subsets in human PBMCs Representative plots showing the gating hierarchy applied to (A) unstimulated and (B) SEB-stimulated PBMCs samples. Singlets were identified using FSC-A versus FSC-H, followed by exclusion of dead cells and dump-positive cells (CD14, CD16, CD19, CD56). Lymphocytes were gated based on FSC/SSC characteristics. CD3^+^ T-cells were subsequently identified and separated into CD4^+^ and CD8^+^ subsets. Regulatory T-cells were defined as CD3^+^ CD4^+^ CD25^+^FoxP3^+^ cells. Memory and homing phenotypes were further characterized based on CD45RA/CD45RO, CCR7, CLA, and CCR9 expression.

This section outlines the gating strategy used to analyze T-cell subsets, regulatory phenotypes, as well as population homing into the skin and gut in flow cytometry data. Data are acquired using BD FACSDiva software (version 8.0.1) and analyzed using FlowJo (version 10.1.0.0). Representative gating is shown in Figure 2 [troubleshooting 5].

53. Analyze data using FlowJo or equivalent software. Gate the samples according to the gating strategy shown in Figure 2.
  a. Exclude Doublets: Eliminate cell clumps by plotting FSC-Height (FSC-H) vs. FSC-Area (FSC-A). Select only the events falling along the diagonal to ensure analysis of individual cells.
  b. Remove Dead Cells and Non-target Lineages: Exclude dead cells and dump-positive cells (CD14, CD16, CD19, CD56) by selecting the negative population in the Viability/Dump channel.
  c. Identify Lymphocytes: Use Side Scatter (SSC) and Forward Scatter (FSC) to gate the lymphocyte population based on size and internal complexity.
  d. Resolve T-cell Subsets: Within the lymphocyte gate, identify the CD3+ T-cell population. From the CD3+ gate, separate cells into CD4+ and CD8+ subsets.
  e. Define CD4+ Tregs and Homing: On the CD4+ gate, identify regulatory T-cells (Tregs) as the CD25+ FoxP3+ double-positive population. Characterize these Tregs further using a cross gate for CLA and CCR9 expression.
  f. Characterize CD8+ Memory and Homing: On the CD8+ population, define memory subsets using CD45RA vs. CD45RO and CCR7. Finally, analyze these subsets for CLA and CCR9 homing markers.

## Expected outcomes

Using this protocol, PBMCs can be reproducibly isolated, cryopreserved, thawed, stimulated, and analyzed by multiparameter flow cytometry. Our post-thaw viability typically ranges from 70% to 90% when freezing and thawing steps are performed correctly. The gating strategy enables clear discrimination of live CD3^+^ T-cells and downstream subsets including CD4^+^ and CD8^+^ subsets. Regulatory T-cells defined as CD4^+^CD25^+^FoxP3^+^ cells are expected to represent approximately 3%–10% of CD4^+^ T-cells, depending on donor characteristics and stimulation conditions. SEB stimulation generally induces robust T-cell activation compared to unstimulated controls, whereas LPS and ZYM often produce more moderate donor-dependent responses. When appropriate fluorescence-minus-one (FMO) and single-stain (SS) controls are used, multicolor staining allows reliable compensation and accurate gating of T-cell subsets, regulatory markers, as well as characterization of homing markers into the skin and gut, and the activation states of the cells.

Inter-individual variability in subset frequencies and activation magnitude is expected, particularly in pediatric clinical samples; however, consistent sample processing enables comparative analysis across stimulation conditions.

## Quantification and statistical analysis

Flow cytometry data are analyzed using FlowJo software (version 10.1.0.0). Cell populations are defined based on sequential gating as described in the Gating/analysis section. Data are reported as the percentage of cells within the indicated gated population. For selected markers, median fluorescence intensity (MFI) was extracted. Statistical analyses were performed as described in the associated research article using GraphPad Prism (version 8.4.3).

## Limitations

This protocol was optimized for the analysis of cryopreserved human PBMCs obtained from pediatric patients with atopic dermatitis and food allergy. Although it can be applied to PBMCs from adult donors (as we already did), differences in cell yield, viability after thawing, baseline distribution of immune cell populations, and responsiveness to stimulation may require adjustment of cell input numbers or stimulation conditions.

Cryopreservation and thawing may differentially affect specific immune cell subsets and surface marker expression. In particular, chemokine receptors and activation markers can be sensitive to temperature fluctuations, sample handling, freeze-thaw cycles, or prolonged *ex vivo* manipulation, potentially influencing their detectability by flow cytometry.^13^ These effects should be considered when comparing freshly isolated and cryopreserved samples or when adapting the protocol to other cohorts.

Flow cytometry data quality depends on proper compensation, gating strategy, and instrument performance. Variations in cytometer configuration, antibody panels, or analysis settings may affect reproducibility across laboratories and may affect direct comparability. In addition, this panel was optimized for a 4-laser BD LSRII; transfer to other instruments may require fluorochrome substitution and renewed antibody titration.

## Troubleshooting

### Problem 1

Reduced cell viability after thawing cryopreserved PBMCs (typically below 70%) (related to Step 23).

Low cell viability after thawing is most commonly caused by fluctuations in storage temperature, incomplete or delayed thawing, prolonged exposure to DMSO, or insufficient removal of DMSO during washing steps. These factors can induce cumulative cellular stress during storage, osmotic stress and membrane damage, resulting in compromised cell integrity and reduced responsiveness to subsequent stimulation and staining procedures.

### Potential solution

• Minimize the time needed for sample transfer. In the case of transport of already cryopreserved PBMCs between facilities, use liquid nitrogen. For the transport from the −150 °C freezer/liquid nitrogen storage to the 37 °C water bath during the thawing and recovery of cryopreserved PBMCs (Step 18) transfer the samples on the dry ice.
• Ensure that the water bath has fully tempered to 37 °C before removing samples from −150 °C freezer/liquid nitrogen (Step 18).
• Avoid temperature fluctuations during storage by minimizing repeated opening of freezers/liquid nitrogen containers and ensuring stable storage conditions.
• Immediately after the thawing proceed with DMSO removal (Step 19).
• When freezing PBMCs (related to Step 12), increase the cell concentration per vial to improve post-thaw recovery and viability if low viability is consistently observed across multiple samples (Step 16).
• As an immediate solution, thaw an additional cryopreserved aliquot of the same sample (Step 18).

### Problem 2

Excessive cell death (typically below 70%) after stimulation due to high concentrations of SEB, LPS, or ZYM (related to Step 26).

Overstimulation can markedly reduce viability after overnight incubation. This is often caused by donor-to-donor variability in cell susceptibility or by errors in preparation of working stimulus concentrations.

### Potential solution

• Verify stock concentrations and dilution calculations before use. Prepare fresh working solutions whenever possible.
• Perform a preliminary titration experiment to determine optimal stimulus concentration for your patient cohort.
• If excessive cell death is observed, reduce stimulus concentration and reassess viability.
• Confirm post-stimulation viability using trypan blue or viability dye before staining to avoid unnecessary antibody use on non-viable samples.

### Problem 3

Suboptimal antibody staining (related to Steps 37 and 44).

Inappropriate antibody dilution can result in weak signal intensity, poor separation between positive and negative populations, or increased nonspecific background staining. This issue is particularly relevant in multiparameter panels combining surface and intracellular markers, where fixation/permeabilization and fluorochrome interactions may alter antibody performance.

### Potential solution

• Perform antibody titration experiments using PBMCs to determine the optimal dilution for each antibody under the exact fixation/permeabilization conditions used in the protocol.
• Verify signal intensity and background levels using SS control and controls.
• Adjust concentrations selectively: If excessive background or signal loss is observed, adjust the concentration of the affected antibody instead of modifying the entire antibody master mix.
• Monitor lot-to-lot variability: Re-titrate antibodies when switching to a new antibody lot or fluorochrome conjugate, as staining performance may vary between batches.

### Problem 4

Compensation artifacts during multicolor acquisition (related to Steps 49–52).

Incorrect compensation setup, or insufficient events in control samples can distort fluorescence spillover correction and compromise identification of rare subsets.

### Potential solution

• Use SS controls prepared on either cells or beads, but do not combine both types in the same automatic compensation procedure.
• Verify detector settings with daily quality-control beads and confirm that no population is off scale in the reference control.
• Use fluorescence-minus-one controls to define gates for dim or overlapping populations.

### Problem 5

Inappropriate detectors voltage settings or inconsistent gating during multicolor flow cytometry analysis (related to Steps 49–53).

Inappropriate detectors voltage settings, fluctuations in flow rate, insufficient event counts, or fluidics disturbances may affect population resolution and lead to inconsistent gating or distorted staining patterns.

### Potential solution

• Acquire sufficient live events at a stable flow rate.
• Reanalyze gating after any adjustment of voltages or antibody titration.
• Use time gate as control step to ensure that the fluidics system is functioning properly by identifying and excluding data collected during periods of non-laminar flow, such as air bubbles or micro-clogs.
• Verify stable instrument performance using daily quality-control procedures before acquisition.

## Resource availability

### Lead contact

Further information and requests for resources and reagents should be directed to and will be fulfilled by the lead contact, Zuzana Jiraskova Zakostelska, Ph.D. (zakostelska@biomed.cas.cz).

### Technical contact

Technical questions regarding execution of this flow cytometry protocol should be directed to and will be fulfilled by the technical contact, Jan Svoboda, Ph.D. (svoboda@biomed.cas.cz).

### Materials availability

This study did not generate new unique materials/reagents.

### Data and code availability

This study did not generate any new code. Information related to data availability should be requested from the lead contact.

## Acknowledgments

This work was supported by grant nos. NU20-05-00038, NW24-07-00042, and NW24-06-00509 from the 10.13039/501100003243Ministry of Health of the Czech Republic (the 10.13039/501100009553Czech Health Research Council) and by the grant “Talking microbes - understanding microbial interactions within One Health framework” (CZ.02.01.01/00/22_008/0004597) from the 10.13039/501100001823Ministry of Education, Youth and Sports of the Czech Republic and the 10.13039/501100004240Academy of Sciences of the Czech Republic.

The authors thank the laboratory technicians at Motol and Homolka University Hospital, especially Jarmila Grecova and Jana Smerdova. The authors also acknowledge the Cytometry and Microscopy Facility at the 10.13039/501100019563Institute of Microbiology of the Czech Academy of Sciences, Prague, CZ, for the access to cytometry equipment.

## Author contributions

Z.J.Z., H.T.-H., D.S., E.K., and A.P. conceived and designed the research. T.T., J.S., Z.S., and Z.J.Z. wrote the manuscript. E.K., A.P., S. Ca., J.C., and A.S. examined the patients and healthy controls and collected samples. Z.J.Z., M.K., T.T., Z.S., S. Co., Z.R., and J.S. performed the experiments and analyzed and interpreted the data. All authors revised and approved the final version of the manuscript.

## Declaration of interests

The authors declare no competing interests.
